# Effect on non-vascular outcomes of lowering LDL cholesterol in patients with chronic kidney disease: results from the Study of Heart and Renal Protection

**DOI:** 10.1186/s12882-017-0545-2

**Published:** 2017-05-01

**Authors:** C. Reith, N. Staplin, W. G. Herrington, W. Stevens, J. Emberson, R. Haynes, M. Mafham, J. Armitage, A. Cass, J. C. Craig, L. Jiang, T. Pedersen, C. Baigent, M. J. Landray, on behalf of the SHARP Collaborative Group

**Affiliations:** 1 Nuffield Department of Population Health (NDPH), University of Oxford, Oxford, UK; 2 Medical Research Council-Population Health Research Unit (MRC-PHRU), NDPH, University of Oxford, Oxford, UK; 3 Menzies School of Health Research, Charles Darwin University, Darwin, Australia; 4 Sydney School of Public Health, University of Sydney, Sydney, Australia; 5 Cardiovascular Institute and Fuwai Hospital, Chinese Academy of Medical Sciences, Beijing, China; 6 Centre of Preventive Medicine, Oslo University Hospital, Oslo, Norway; 7 Faculty of Medicine, University of Oslo, Oslo, Norway

## Abstract

**Background:**

Reducing LDL cholesterol (LDL-C) with statin-based therapy reduces the risk of major atherosclerotic events among patients with chronic kidney disease (CKD), with no evidence of an excess risk of cancer or death from any non-vascular cause. However, non-randomized data have suggested that statin therapy may have effects (both adverse and beneficial) on particular non-vascular conditions that do not cause death.

**Methods:**

The Study of Heart and Renal Protection (SHARP) randomized patients with CKD to simvastatin 20 mg plus ezetimibe 10 mg (simvastatin/ezetimibe) daily versus matching placebo. Participants were followed up at least 6 monthly and all post-randomization serious adverse events (SAEs) were recorded. This supplementary analysis reports the effects of treatment on non-vascular SAEs, overall, by system of disease, by baseline characteristics, and by duration of follow-up.

**Results:**

During a median of 4.9 years follow-up, similar numbers of participants in the two groups experienced at least one non-vascular SAE (3551 [76.4%] simvastatin/ezetimibe vs 3537 [76.6%] placebo; risk ratio [RR] 0.99, 95% confidence interval [CI] 0.95–1.04). There was no good evidence of any significant effect of simvastatin/ezetimibe on SAEs attributed to any particular nonvascular disease system (of 43 comparisons, only 3 yielded an uncorrected *p* value < 0.05,﻿ ﻿of which﻿﻿ ﻿the smallest was *p* = 0.02). The relative risk of any nonvascular SAE did not vary significantly among particular prognostic subgroups or by duration of follow-up.

**Conclusions:**

In the SHARP trial, allocation to simvastatin/ezetimibe combination therapy was not associated with any significant non-vascular hazard.

**Trials registration:**

SHARP was retrospectively registered after the first participant was enrolled in 2003 at ISRCTN (ISRCTN54137607 on 31 January 2005: http://www.isrctn.com/ISRCTN54137607) and ClinicalTrials.gov (NCT00125593 on 29 July 2005: https://clinicaltrials.gov/ct2/show/NCT00125593).

**Electronic supplementary material:**

The online version of this article (doi:10.1186/s12882-017-0545-2) contains supplementary material, which is available to authorized users.

## Background

Meta-analyses of individual participant data from large randomized controlled trials have shown that statin therapy reduces the risk of major vascular events (defined as myocardial infarction (MI), coronary death, stroke or coronary revascularization) by about one fifth per mmol/L (40 mg/dL) reduction in low-density lipoprotein cholesterol (LDL-C), without any increase in the risk of non-vascular causes of death or of site-specific cancer [1–3]. Benefits have been demonstrated in a wide range of people with pre-existing vascular disease and diabetes [4], as well as in those with no prior history of vascular disease [5].

Chronic kidney disease (CKD) is associated with a significantly increased risk of cardiovascular disease (CVD), with premature CVD being a leading cause of death in people with CKD [6]. Several randomized placebo-controlled trials have tested the effects of lowering LDL-C with statin-based therapy in patients with CKD [7–9]. The Study of Heart and Renal Protection (SHARP) was the largest such trial, being conducted among over 9400 patients. In SHARP, allocation to the combination of simvastatin 20 mg plus ezetimibe 10 mg (simvastatin/ezetimibe) reduced major atherosclerotic events (MAEs), defined as non-fatal MI or coronary death, non-haemorrhagic stroke, or any arterial revascularization procedure, by 17% (95% confidence interval [CI] 6–26%; *p* = 0.0021) [9]. This reduction was achieved without any significant increase in any of the prespecified safety outcomes [10] of: muscle pain; elevation of creatine kinase (CK) to five to ten times the upper limit of normal (ULN) or greater than ten times the ULN; complications of gallstones and persistent elevation of liver transaminases to greater than three times the ULN. There were very few cases of the pre-specified outcome of myopathy (9 [0.2%] simvastatin/ezetimibe vs 5 [0.1%] placebo) or of more severe cases of rhabdomyolysis (4 [0.1%] simvastatin/ezetimibe vs 1 [0.0%] placebo), and there was no significant excess risk of cancer or of death from any non-vascular cause [9].

In populations without CKD, large randomized trials, and meta-analyses of those trials, have shown that statins cause small increases in the risk of myopathy [11–13], diabetes [13–15], and probably haemorrhagic stroke [2, 13, 16]. However, reports from non-randomized observational studies (which are susceptible to bias) have also suggested that statin use is associated with higher rates of a wide range of other adverse events, including hepatic dysfunction [17, 18], acute kidney injury [17, 19], impaired cognition [20] and sleep disturbance [21]. Conversely, there have also been reports from such studies of associations between statin use and lower rates of some non-vascular events, including respiratory infections [22, 23], gastrointestinal bleeding [24], Parkinson’s disease [25, 26] and fractures [27].

Patients with CKD are typically at higher risk of non-vascular events than the general population due to their potential for comorbid disease in association with renal impairment [28], hence it is important to assess whether statin-based therapy yields increases or decreases in the risks of other types of outcome. Such an assessment is most reliably achieved by analysis of large-scale randomized trials, and the aim of the present paper is to conduct such analyses in the SHARP trial, in which all serious adverse events (SAEs) were collected routinely at 6 monthly visits for a median of about 5 years in a wide range of patients with CKD who were distributed among 18 countries worldwide.

## Methods

Details of the SHARP trial objectives, design, and methods have been reported previously in accordance with the CONSORT guidelines [9, 10]. The SHARP trial was carried out in accordance with the Declaration of Helsinki.

### Recruitment

People aged 40 years and older were eligible to participate if they had CKD with more than one previous measurement of serum or plasma creatinine of at least 150 μmol/L (1.7 mg/dL) in men or 130 μmol/L (1.5 mg/dL) in women, or were receiving maintenance dialysis. Participants with prior MI or coronary revascularization were excluded. Potentially eligible participants attended a screening visit at which medical history, including history of diabetes, was recorded and written informed consent obtained. After 6 weeks of placebo run-in, participants who remained willing and eligible, had taken at least 90% of the run-in treatment, and who were thought likely to be able to attend study clinics for at least 4 years were randomized in a 4:4:1 ratio to simvastatin 20 mg plus ezetimibe 10 mg daily versus matching placebo combination therapy versus simvastatin 20 mg alone. Participants who were allocated simvastatin only were re-randomized after one year to simvastatin/ezetimibe vs placebo combination therapy.

### Follow-up and recording of SAEs

After initial randomization, participants were followed-up in study clinics at 2 and 6 months and then every 6 months for at least 4 years. At each of these visits, information was recorded on all SAEs (defined as any untoward medical occurrence that results in death; is life-threatening; requires inpatient hospitalization or results in prolongation of existing hospitalization; results in persistent or significant disability/incapacity; is a congenital anomaly/birth defect, or; is a medically important event or reaction [with the SHARP protocol specifying cancers, myopathy, rhabdomyolysis, cholecystectomy or complications of gallstones and hepatitis as events of particular relevance]) occurring since the last visit. Current co-medication was also recorded at all visits. The development of diabetes mellitus among participants without diabetes at baseline (a tertiary endpoint) was defined as an SAE due to diabetes or the initiation of diabetic medications in participants not known to have diabetes mellitus at randomization.

If a participant became unwilling or unable to attend the follow-up visits, information about SAEs was obtained from them (or their relative or carer) by telephone or from their own doctors until the scheduled end of the study. Local study staff then sought additional information from hospital records and other appropriate sources about all reports of SAEs that might relate to study outcomes (ie, death, MI, cardiac arrest, angina, heart failure, stroke, transient ischaemic attack, revascularization procedures, angiography, amputation, initiation of dialysis, kidney transplant, renal failure, cancer, myopathy, rhabdomyolysis, hepatobiliary or pancreatic conditions). This information was sent to the international coordinating centre for central adjudication, in accordance with pre-specified definitions, by trained clinicians who were masked to study treatment allocation. However, the majority of non-vascular outcomes were not adjudicated.

### Statistical analysis

All analyses reported here are post-hoc. Intention-to-treat analyses assessed the effect of allocation to simvastatin/ezetimibe on time to first SAE, with subdivision of analyses into vascular SAE (MAEs, as defined a priori [9, 10], plus any other vascular SAE) and non-vascular SAEs (see Additional file 1). Subsidiary analyses subdivided analyses of non-vascular SAEs by system of disease (cancer, renal, respiratory, hepatobiliary, other gastrointestinal, other medical causes, and trauma/fracture) by outcome (fatal versus non-fatal), by baseline characteristics reported previously (age, sex, prior diabetes, baseline LDL-C, body mass index [BMI], and renal status) and by baseline characteristics not previously reported (ethnicity). Further analyses explored the effect on non-vascular SAEs by duration of follow-up.

Standard log-rank methods, stratified by whether participants were initially randomized to simvastatin only or not, were used to provide estimates of average event rate ratios (RRs), associated 95% CIs and 2-sided *p*-values, from the time of randomization to simvastatin/ezetimibe versus placebo (ie, ignoring the 168 participants who were initially randomized to simvastatin 20 mg only and not subsequently re-randomized to simvastatin/ezetimibe versus placebo). Standard χ^2^ tests for heterogeneity (or, where appropriate, trend) were used to compare event rate ratios between subgroups. While no formal adjustment was made for the p-values, due allowance for multiple hypothesis-testing and the post-hoc exploratory nature of these analyses were made when interpreting the results. Analyses were conducted using SAS version 9.3 and R version 2.14.2.

## Results

Nine thousand two hundred seventy participants were randomly assigned to simvastatin/ezetimibe (4650 participants) or placebo (4620 participants), with good balance achieved in measured characteristics (Table 1). Mean age at randomization was 62 years (standard deviation [SD] 12), 5800 (63%) were male, 1393 (15%) had a history of vascular disease, 2094 (23%) had diabetes and mean baseline non-fasting directly measured LDL-C was 2.8 (SD 0.9) mmol/L. The majority of participants were of white ethnicity (72%), but a substantial minority of participants were Chinese (12%) or of other Asian ethnicity (10%). Approximately one-third of participants were receiving maintenance dialysis at randomization. The median duration of follow-up among survivors was 4.9 years.

**Table 1:** Baseline demographic and laboratory measurements by treatment allocation

	Simvastatin plus ezetimibe (*n* = 4650)	Placebo (*n* = 4620)
Demographics		
Age at randomization (years)^a^	62 (12)	62 (12)
Men	2915 (63%)	2885 (62%)
Ethnicity		
White	3332 (72%)	3314 (72%)
Black	137 (3%)	127 (3%)
Chinese	557 (12%)	563 (12%)
Other Asian	486 (10%)	480 (10%)
Other/not specified	138 (3%)	136 (3%)
Prior disease		
Prior vascular disease^a^	711 (15%)	682 (15%)
Diabetes^a^	1054 (23%)	1040 (23%)
Renal status		
On dialysis^a^	1534 (33%)	1491 (32%)
Haemodialysis	1275 (27%)	1253 (27%)
Peritoneal dialysis	259 (6%)	238 (5%)
Not on dialysis^a^	3116 (67%)	3129 (68%)
Baseline measurements		
Total cholesterol (mmol/L)^a^	4.88 (1.20)	4.90 (1.17)
LDL cholesterol (mmol/L)^a^	2.77 (0.88)	2.78 (0.87)
HDL cholesterol (mmol/L)^a^	1.12 (0.35)	1.11 (0.34)
Triglycerides (mmol/L)^a^	2.31 (1.76)	2.34 (1.68)
Body mass index (kg/m^2^)^a^	27.1 (5.7)	27.1 (5.6)
Renal function		
MDRD-estimated GFR (mL/min/1.73 m^2^)^a,b,c^	26.6 (12.9)	26.6 (13.1)
≥60	44 (1%)	44 (1%)
≥30 to <60	1100 (37%)	1055 (35%)
≥15 to <30	1246 (41%)	1319 (44%)
< 15	613 (20%)	606 (20%)
Not available	113	105

Data are n (%) or mean (SD)

*MDRD* Modified Diet in Renal Disease, *GFR* glomerular filtration rate

^a^Variables updated at 1 year for patients originally allocated simvastatin only who were rerandomized to simvastatin plus ezetimibe or placebo

^b^Percentages exclude participants for whom data were not available for that category

^c^For patients not on dialysis

### Serious adverse events by system of disease and event outcome

**Fig. 1:**
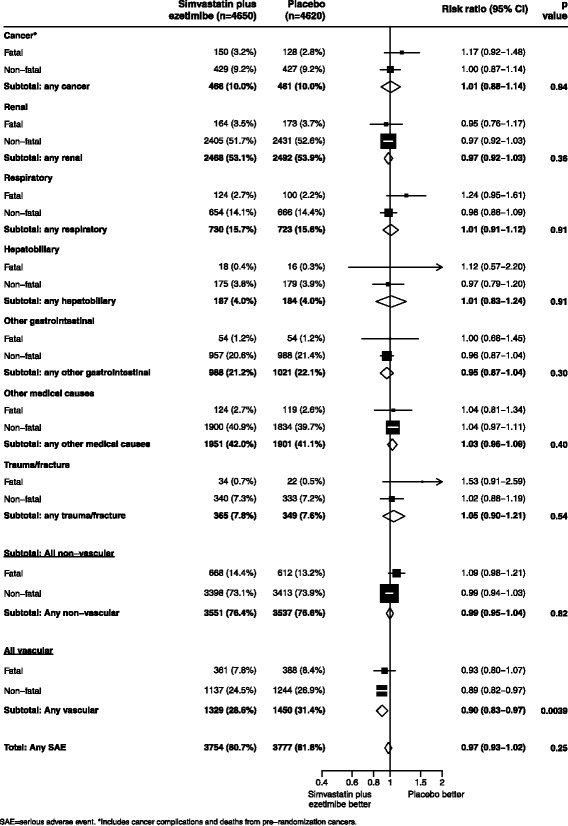
All SAEs, by system of disease and outcome

**Fig. 2:**
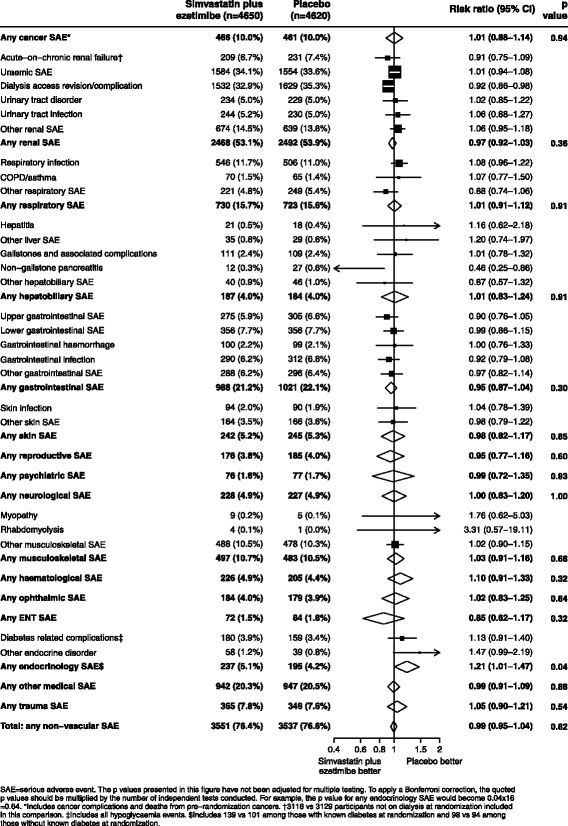
All non−vascular SAEs, by system of disease with sub−categories

Allocation to simvastatin/ezetimibe resulted in a significant reduction in any vascular SAE (1329 [28.6%] simvastatin/ezetimibe vs 1450 [31.4%] placebo; RR 0.90, 95% CI 0.83–0.97; Fig. 1). There was no significant effect on the proportion experiencing at least one non-vascular SAE (3551 [76.4%] simvastatin/ezetimibe vs 3537 [76.6%] placebo; RR 0.99, 95% CI 0.95–1.04; *p* = 0.82), and no significant effect on fatal or non-fatal non-vascular SAEs when subdivided into disease systems (Fig. 1). Taking all non-vascular SAEs together (fatal and non-fatal) and ﻿ignoring any correction for mutiple testing, allocation to simvastatin/ezetimibe was associated with an increased risk of endocrine SAEs (237 [5.1%] vs 195 [4.2%]; RR 1.21, 95% CI 1.01–1.47), but closer examination of these endocrine SAEs by subdivision into hormonal systems revealed no significant evidence of hazard for any individual category of events: diabetes-related complications (180 [3.9%] vs 159 [3.4%]; RR 1.13, 95% CI 0.91–1.40), thyroid-related conditions (44 [0.9%] vs 28 [0.6%]; RR 1.55, 95% CI 0.98–2.46), adrenal disorders (5 [0.1%] vs 4 [0.1%]; RR 1.24, 95% CI 0.34–4.60) and other endocrine disorders (10 [0.2%] vs 8 [0.2%]; RR 1.24, 95% CI 0.49–3.13). There were nominally fewer cases of non-gallstone pancreatitis among participants allocated simvastatin/ezetimibe (12 [0.3%] vs 27 [0.6%]; RR 0.46, 95% CI 0.25–0.86; *p* = 0.02), and a reduction in dialysis access revisions/complications (1532 [32.9%] vs 1629 [35.3%]; RR 0.92, 95% CI 0.86-0.98)﻿, but no other apparent differences for other non-vascular SAEs (Fig. 2). Among 7176 participants without diabetes at baseline, 172 [4.8%] in the simvastatin/ezetimibe group vs 162 [4.5%] in the placebo group developed new-onset diabetes (RR 1.06, 95% CI 0.86–1.32).

### Non-vascular serious adverse events by baseline characteristics

When participants were subdivided by baseline characteristics, there was no evidence of any heterogeneity (or, for a variable that could be ordered, trend) in the effect of simvastatin/ezetimibe on non-vascular SAEs (Additional file 2: Figure S1). In particular there was no evidence of heterogeneity in the effect of simvastatin/ezetimibe on non-vascular SAEs either overall or by system of disease between patients on dialysis at randomization and those not (Additional file 2: Figure S2).

### Non-vascular serious adverse events by duration of follow-up

There was no evidence of any trend toward an increasing (or decreasing) risk ratio for non-vascular SAEs with increasing duration of follow-up, either for non-vascular death (trend *χ*_1_^2^ = 1.69; *p* = 0.19) or for non-fatal non-vascular SAEs (trend *χ*_1_^2^ = 0.05; *p* = 0.83) (Additional file 2: Figure S3).

## Discussion

In SHARP, simvastatin/ezetimibe resulted in a significant reduction in MAEs amongst participants with moderate to advanced CKD, with no excess risk of any of the prespecified safety outcomes [9, 10]. The present subsidiary analyses indicate that treatment was not associated with an overall excess risk of non-vascular SAEs, and nor was there any evidence that non-vascular SAEs were increased among particular subgroups of participant defined by baseline characteristics including age, sex, diabetes, baseline LDL-C, BMI, ethnicity, and renal status.

Despite the addition of pancreatitis to the post-marketing experience section of the simvastatin/ezetimibe drug label as a potential undesirable effect [29], we observed, if anything, a reduction in non-gallstone pancreatitis events among those assigned to simvastatin/ezetimibe in SHARP, but the relevance of our findings is unclear due to the small numbers of events. ﻿A﻿ reduction in dialysis access rev﻿isions or co﻿mplications was also observed, but in previous exploratory analyses, this finding was not confirmed in data from the AURORA trial, suggesting that any benefits of lowering LDL-C on vascular access patency are likely to be modest [30].

Allocation to simvastatin/ezetimibe in SHARP was associated with a marginally increased risk of endocrine SAEs overall (237 [5.1%] vs 195 [4.2%]; RR 1.21, 95% CI 1.01-1.47) but such a difference is also consistent with the play of chance given the number of tests performed. Moreover, closer examination of these endocrine SAEs by subdivision into hormonal systems revealed no significant evidence of hazard.

Meta-analyses of randomized trials have shown that statin therapy is associated with approximately a 10 to 20% proportional increase in the risk for developing diabetes, equating to approximately 1 to 2 additional cases per 1000 person-years of statin treatment in those trials [13–15]. This increased risk is thought to be related to LDL receptor-mediated transmembrane cholesterol transport [31], and is supported by evidence from Mendelian randomization studies of various LDL-C targets [32–34]. The observed non-significant 6% excess risk of diabetes seen in SHARP was consistent with these previous results, although there was limited power to assess whether the treatment effect differed from that observed in previous trials of a statin.

Some observational studies have found an inverse relationship between cholesterol levels and infectious disease [35, 36], leading some to suggest that lowering LDL-C may be harmful. In contrast, other studies have postulated that statins are potentially protective against infection, particularly respiratory infections [22, 23, 37], whilst randomized controlled trials in sepsis-associated acute respiratory distress syndrome and in chronic obstructive pulmonary disease have demonstrated no therapeutic benefit [38–40]. In SHARP, there were no significant effects of simvastatin/ezetimibe on the risk of infection (urinary tract, respiratory, gastrointestinal or skin). The inverse relationship between cholesterol levels and infectious disease seen in observational studies may therefore reflect reverse causality, since people with CKD are often sick and malnourished (and hence have a lower LDL-C) and are also more prone to infections.

Studies of statins have postulated both protective effects for statins against renal progression [41] and possible nephrotoxic effects [17, 19, 42]. Previously reported analyses from SHARP showed no significant reductions in any of the pre-specified measures of renal disease progression (end-stage renal disease defined as commencement of maintenance dialysis or transplantation) [9], and exploratory analyses showed no effect on urinary albumin creatinine ratio at 2.5 years [43]. Thus whilst approximately 7% of participants not on dialysis at randomization developed acute on chronic renal failure, there was no evidence of an increased risk with simvastatin/ezetimibe.

A strength of the analyses described in this paper is that they are based on randomized assessment of treatment effects as opposed to inference from observational data (which can be subject to bias) [13]. However, a limitation is that they are only based on an average of five years’ follow-up, so that longer-term effects cannot be quantified. Long-term follow-up of efficacy and safety in randomized trials of statins in other populations has demonstrated continuing benefits on vascular events and reassuring safety for non-vascular events (including cancer) [44, 45]. Such post-trial follow-up is therefore now underway in the SHARP cohort. Furthermore, although SHARP is the largest randomized trial in CKD patients to date, it lacks statistical power to examine rare events or certain non-vascular events in detail (such as diabetes). The reliable assessment of any such non-vascular effects is best done through large-scale meta-analyses, such as those which will be conducted by the Cholesterol Treatment Trialists’ Collaboration [46]. Another possible limitation of these analyses is that the majority of non-vascular outcomes were not verified by clinician adjudicators. However, similar intention-to-treat analyses of non-adjudicated, non-vascular SAEs have previously been used to demonstrate both known and previously unrecognised hazards of niacin in the large-scale HPS2-THRIVE trial [47].

## Conclusions

In SHARP, simvastatin/ezetimibe did not result in any significant adverse effect on non-vascular events during a median of about 5 years’ treatment among patients with CKD. These findings add to the safety information from the IMPROVE-IT trial which compared simvastatin/ezetimibe to simvastatin monotherapy in approximately 18,000 participants with acute coronary syndrome, and which therefore assessed the safety of ezetimibe when added to statin therapy. IMPROVE-IT reported no meaningful differences between the treatment groups in adverse events (including cancer, non-vascular mortality and muscle, gallbladder, hepatic and new onset diabetes adverse events) [48, 49]. The results of SHARP and IMPROVE-IT taken together therefore indicate that adding ezetimibe to simvastatin is both effective and generally well-tolerated.

## Additional files

Additional file 1: SHARP event code categories. (DOCX 31 kb)

Additional file 2: Figure S1. All non-vascular SAEs, by particular baseline characteristics. **Figure S2.** All non-vascular SAEs, by system of disease and dialysis status. **Figure S3.** All non-vascular SAEs, by duration of follow-up. (PDF 21 kb)

## Abbreviations

BMI: Body mass index
CI: Confidence interval
CK: Creatine kinase
CKD: Chronic kidney disease
CONSORT: CONsolidated Standards of Reporting Trials
CVD: Cardiovascular disease
GFR: Glomerular filtration rate
HPS2-THRIVE: Heart Protection Study2-Treatment of HDL to Reduce the Incidence of Vascular Events
IMPROVE-IT: Improved Reduction of Outcomes: Vytorin Efficacy International Trial
ISCRTN: International Standard Randomised Controlled Trial Number
LDL-C: Low density lipoprotein cholesterol
MAE: Major atherosclerotic event
MDRD: Modified Diet in Renal Disease
MI: Myocardial infarction
RR: Rate ratio
SAE: Serious adverse event
SAS: Statistical Analysis System
SD: Standard deviation
SHARP: Study of Heart and Renal Protection
ULN: Upper limit of normal
