# Supplementary material for: Effect on non-vascular outcomes of lowering LDL cholesterol in patients with chronic kidney disease: results from the Study of Heart and Renal Protection
Source: BMC Nephrol. 2017 May 1;18:147. doi: 10.1186/s12882-017-0545-2 (PMC5412040; doi:10.1186/s12882-017-0545-2)
Supplement: Supplementary file 1 — SHARP event code categories. (DOCX 31 kb) [file 12882_2017_545_MOESM1_ESM.docx]

| ***Non-vascular SAEs*** |  |
| --- | --- |
| **Cancer** |  |
| Any cancer (including complications) | Basal cell carcinoma/rodent ulcer; bladder cancer; breast cancer; cancer in renal transplant; cancer of lip/nasopharynx/pharynx/mouth; cancer of the rectum or anus; carcinoid syndrome/carcinoid tumour; carcinomatosis/secondary metastases (any site); cervical cancer; chemotherapy; chronic lymphocytic leukaemia (CLL); colon cancer; complication or treatment of cancer; gall bladder cancer/bile duct cancer/cholangiocarcinoma; Hodgkin's lymphoma; large bowel cancer; laryngeal cancer/cancer of the larynx; leukaemia (any cell type); liver cancer/hepatocellular carcinoma/intra-hepatic duct cancer; lung cancer; lymphoma; malignant brain tumour/brain cancer; malignant melanoma; mesothelioma; myeloma; non-hodgkin's lymphoma; non-melanoma skin cancer; oesophageal cancer; ovarian cancer; pancreas cancer; penis cancer; pre-randomization cancer; prostate cancer; radiotherapy; recurrence of already reported cancer; renal cell carcinoma/hypernephroma/kidney cancer; salivary gland cancer; sarcoma/fibrosarcoma or dermato-fibrosarcoma; small intestine cancer/small bowel cancer; spinal tumour; squamous cell carcinoma; squamous cell carcinoma-in-situ; stomach cancer; symptoms from already reported cancer; testicular cancer; thyroid cancer; uterine/endometrial cancer/cancer of uterus (body); vulval cancer |
|  |  |
| **Renal** |  |
| Acute-on-chronic renal failure | Acute on chronic renal failure; acute on chronic renal failure not requiring dialysis; acute on chronic renal failure requiring dialysis |
| Uraemic SAE | Cadaveric renal transplantation; change of dialysis modality; conservative care for ESRD; initiation of dialysis; initiation of haemodialysis; initiation of peritoneal dialysis; living donor renal transplantation; renal transplantation; simultaneous kidney/pancreas transplantation; uraemia; withdrawal of dialysis |
| Dialysis access revision/complication | Bleeding from arteriovenous fistula/graft; blocked peritoneal dialysis catheter; blocked/thrombosed arteriovenous fistula/graft; creation of permanent arteriovenous fistula; creation of synthetic graft for dialysis; failed haemodialysis because of vascular access problem; fistulogram; infected arteriovenous fistula/graft; insertion of peritoneal dialysis catheter; insertion of temporary venous line; insertion of tunnelled venous line; localised dialysis catheter infection/abscess; localised peritoneal dialysis catheter infection/abscess; percutaneous angioplasty/embolectomy of arteriovenous fistula/graft; peritoneal dialysis associated peritonitis; peritoneal dialysis catheter procedure; removal arteriovenous fistula/graft; removal of haemodialysis catheter; removal of peritoneal dialysis catheter; sclerosing peritonitis; sepsis secondary to dialysis catheter infection; surgical exploration/repair arteriovenous fistula/graft; venoplasty of central venous stenosis |
| Urinary tract disorder | Bladder biopsy/cystoscopy with biopsy; bladder investigations; bladder problem (except cancer); bladder surgery; bladder tumour/warts; cystectomy; cystoscopy or urethroscopy; ileal conduit surgery; insertion or removal trans-urethral or supra-pubic catheter; kidney stone/ureter stone/bladder stone; nephrostomy+/-stent/removal of renal stent; percutaneous or open kidney stone surgery/lithotripsy; renal colic or kidney pain; transurethral resection of bladder tumour (TURBT); ureteric obstruction/hydronephrosis; ureteric surgery; urethral stricture/dilatation/surgery; urinary retention |
| Urinary tract infection | Urinary tract infection (UTI)/kidney infection |
| Other renal SAE | Acute immunosuppressive medication toxicity; acute renal transplant dysfunction; acute renal transplant rejection; calciphylaxis; chronic allograft nephropathy; chronic immunosuppressive medication toxicity; chronic renal transplant rejection; chronic renal transplant dysfunction; fluid overload; haematuria; hypercalcaemia; hyperkalaemia; hypocalcaemia; hypokalaemia; hypotension on dialysis; kidney investigations/procedure; native kidney or renal transplant biopsy; native nephrectomy; nephrectomy; nephrotic syndrome; parathyroid gland problem; parathyroid hyperplasia/adenoma; parathyroidectomy; polycystic kidney disease; post renal transplant urinary sepsis; recurrence of primary renal disease in renal transplant; renal transplant complication; transplant nephrectomy; transplant renal artery angioplasty +/- stent |
| **Respiratory** |  |
| Respiratory infection | Aspiration pneumonia/pneumonitis; bronchiectasis; cough with fever/chest infection/bronchitis; empyema; lung or tracheal abscess; pneumonia |
| COPD/asthma | Acute exacerbation of asthma; acute exacerbation of chronic obstructive airways disease (COAD/COPD); emphysema |
| Other respiratory SAE | Asbestosis; atelectasis/collapse of lung/pulmonary collapse; bronchoscopy +/- biopsy; disorder of pleura and pleural cavity; dyspnoea/shortness of breath; fibrosing alveolitis/alveolitis unspecified; haemoptysis/coughing blood; haemothorax; interstitial lung disease; lung removal/pneumonectomy/lobectomy; lung/pleural biopsy; pleural effusion; pleurectomy/ pleuradhesis/ pneumothorax surgery; pleurisy/pleuritic chest pain; pneumothorax; pulmonary fibrosis; respiratory arrest; respiratory failure; respiratory investigations; sarcoidosis; sleep apnoea; sleep study; stridor/airway obstruction; thoracotomy/chest or lung surgery; tonsil/adenoid disease; tonsillectomy |
| **Hepatobiliary** |  |
| Infectious hepatitis | Acute hepatitis B; acute hepatitis C; CMV hepatitis; other acute viral hepatitis; alcoholic hepatitis; other specified inflammatory liver disease; toxic liver disease NOS; toxic liver disease with acute hepatitis; toxic liver disease with cholestasis; toxic liver disease with hepatitis, not elsewhere classified; hepatitis; non infective hepatitis |
| Other liver SAE | Alcoholic cirrhosis; alcoholic liver disease; chronic hepatitis B; chronic hepatitis C; cirrhosis NOS; cirrhosis/chronic liver disease; fatty liver NEC; hepatic veno-occlusive disease; jaundice; liver abscess; liver biopsy; liver failure; liver infarction; portal hypertension; primary biliary cirrhosis |
| Gallstones and associated complications | Acute pancreatitis (gallstones); bile duct stone with cholangitis; bile duct stone with cholecystitis; bile duct stone without cholecystitis or cholangitis; cholecystectomy/gall bladder surgery; cholelithiasis/gall stones; gallstones with acute cholecystitis; gallstones with other cholecystitis; other cholelithiasis |
| Non-gallstone pancreatitis | Acute pancreatitis (alcohol); acute pancreatitis (drug-induced); acute pancreatitis (idiopathic); acute pancreatitis (other); chronic pancreatitis; chronic pancreatitis (alcohol) |
| Other hepatobiliary SAE | Bile duct disease; bile duct surgery; biliary obstruction/ ascending cholangitis; cholecystitis; endoscopic retrograde cholangio-pancreatogram (ERCP); gall bladder disease; liver surgery; liver transplant; liver/pancreatic/biliary surgery; other disease of gall bladder; pancreas surgery; pancreas transplant; pseudocyst of pancreas; splenectomy |
|  |  |
| **Other gastrointestinal** |  |
| Upper gastrointestinal SAE | Biopsy/removal oesophageal lesion; biopsy/removal small bowel lesion; biopsy/removal stomach lesion; difficulty swallowing/dysphagia/swallowing symptoms; duodenal ulcer; duodenitis; endoscopic treatment of varices/sclerotherapy/banding; endoscopy of upper GI tract/gastroscopy; gastrectomy; gastric erosions; gastric or duodenal ulcer surgery; gastric/stomach ulcer; gastritis (any type); gastro oesophageal reflux (GORD)/hiatus hernia symptoms; oesophageal dilatation +/-stent; oesophageal disorder; oesophageal perforation; oesophageal stricture/obstruction of the oesophagus; oesophageal varices; oesophagitis/Barrett's oesophagus; oesophago-gastrectomy; percutaneous endoscopic gastrostomy (PEG); stomach disorder; stomach surgery; upper gastrointestinal surgery |
| Lower gastrointestinal SAE | Anterior resection/abdomino-perineal resection; appendicectomy; appendicitis; biopsy/removal large bowel lesion; bowel perforation/perforated diverticulum; colectomy /hemicolectomy; colonoscopy; colostomy fashioning/refashioning/reversal/problem; constipation; diverticular disease/diverticulitis; haemorrhoidectomy/piles surgery/banding of haemorrhoids; haemorrhoids/piles; large bowel problem; large bowel resection; pelvic abscess/drainage of; rectal or anal bleeding; rectal or anal surgery; rectal or colon adenoma/villous adenoma; rectal or colon polypectomy; rectal prolapse; rectal/perianal abscess/drainage of; sigmoidoscopy or proctoscopy |
| Gastrointestinal haemorrhage | Gastrointestinal haemorrhage |
| Gastrointestinal infection | Abdominal distension/swelling; abdominal pain; ascites/drainage of ascites; diarrhoea; diarrhoea & vomiting/food poisoning/gastroenteritis; infective colitis; nausea/vomiting |
| Other gastrointestinal SAE | Abdominal cavity hernia (symptoms not repair); abdominal surgery; biopsy GI tract; bowel infarction; bowel problem; bowel resection; bowel surgery; bowel/intestinal obstruction; colitis; Crohn's disease; diagnostic laparoscopy; gastrointestinal investigations/procedure; hernia repair; hiatus hernia surgery; ileostomy fashioning/refashioning/reversal/problem; incisional hernia repair; inflammatory bowel disease; inguinal/femoral hernia repair; intra-abdominal adhesions; irritable bowel syndrome; laparotomy; malabsorption/coeliac disease; parastomal/intra-abdominal abscess/drainage of; peritonitis (not associated with peritoneal dialysis); small bowel problem; small bowel resection; small bowel surgery; ulcerative colitis; umbilical hernia repair |
| **Other medical causes** |  |
| Any skin SAE |  |
| Skin infection | Boil/skin abscess/drainage of abscess; cellulitis; discharging skin sinus/sinus to skin/pilonidal sinus; infected (lymph) gland/lymphadenitis; infected toe/infected finger/infected foot or hand; skin infection |
| Other skin | Blistering/desquamating skin disorder; chronic skin ulcer; debridement of skin/subcutaneous tissue; eczema/dermatitis; excision of lipoma/cyst; ingrowing toenail/toenail infection; leg or foot ulcer; lipoma; melanoma-in-situ; pemphigoid; pemphigus; pressure sores/decubitus ulcer; psoriasis; rash; removal/biopsy of skin/subcutaneous lesion; sebaceous cyst/skin cyst; seborrhoeic keratosis/seborrhoeic wart; skin disorder (except cancer); skin graft; skin lump; skin operation or plastic surgery; solar keratosis; squamous cell carcinoma-in-situ |
| Any reproductive SAE | Biopsy of the breast; biopsy of the prostate; breast disease (except cancer); breast surgery; cervical biopsy/cone biopsy; circumcision; colposcopy; dilatation of the cervix and curretage of the uterus (D&C); endometrial ablation; epididymo-orchitis/testicular infection; gynaecological investigations; hydrocoele/hydrocoele operation or drainage; hysterectomy (any type) with/without oophorectomy; hysteroscopy; intrauterine contraceptive device insertion/removal; lumpectomy of breast; mammoplasty/cosmetic operation on breast; mastectomy; oophorectomy; orchidectomy/removal of testicle; ovarian cyst; ovarian lump/lesion removal; paraphimosis/phimosis; penis problem; penis surgery; prostate investigations; prostate operation/prostatectomy; prostate problem (except cancer); radical prostatectomy; removal of epididymal cyst or scrotal cyst; scrotal/groin/testicular abscess; sterilisation; suspension of uterus/colposuspension; testicular operation; testicular problem/pain/swelling; transurethral resection of prostate (TURP); uterine biospy or polypectomy; uterine fibroids; vaginal bleeding/PV bleed; vaginal prolapse; vaginal prolapse repair /pelvic floor repair/bladder repair in women; varicocoele/varicocoele surgery; vasectomy/reversal of vasectomy; vulval biopsy/removal of vulval lesion |
| Any psychiatric SAE | Alcohol and/or substance misuse; alcohol/drug detoxification; anxiety/panic attacks; attempted suicide; deliberate self-harm; dementia/cognitive impairment; depression; nervous breakdown; overdose; psychological/psychiatric problem; schizophrenia; self-inflicted injury/self-mutilation |
| Any neurological SAE | Acute infective polyneuritis/Guillane Barré/Miller Fisher syndrome; bacterial meningitis; Bell's (facial) palsy; benign brain tumour; brain abscess/intracranial abscess; brain surgery; carpal tunnel release operation/procedure; carpal tunnel syndrome; cerebroventricular shunt/shunt for hydrocephalus; chronic/degenerative neurological disorder; confusion or disorientation; cranial nerve problem/palsy; encephalitis; fit/ convulsion (not known epilepsy); fit/convulsion in known epileptic; headache; intracranial haematoma drainage; loss of consciousness/blackout; meningitis; migraine; motor neurone disease; multiple sclerosis/demyelination; neurological investigations; neurological pain/neuralgia; neuroma; neurosurgery; other neurological problem; paraplegia; Parkinson's disease; peripheral nerve disorder; peripheral nerve surgery/biopsy; peripheral neuropathy/neuropathy; raised intracranial pressure (including papilloedema); seizure/fit/convulsion; spinal cord disorder; spinal cord surgery; trapped nerve/compressed nerve; viral meningitis |
| Any musculoskeletal SAE |  |
| Myopathy | CK 10-40x no end organ damage, asymptomatic; CK 10-40x no end organ damage, muscle symptoms; CK 10-40x on dialysis, asymptomatic; CK 10-40x on dialysis, muscle symptoms; CK 10-40x, end organ damage; myopathy (drug induced); myopathy (infection); myopathy (other cause); myopathy (trauma); myopathy (uncertain cause) |
| Rhabdomyolysis | CK >40x no end organ damage, asymptomatic; CK >40x no end organ damage, muscle symptoms; CK >40x on dialysis, asymptomatic; CK >40x on dialysis, muscle symptoms; CK >40x with end organ damage |
| Other musculoskeletal SAE | Acute or chronic osteomyelitis; ankle joint surgery; ankylosing spondylitis; arthritis/arthropathy; arthroscopy; back pain or problem; bakers cyst; bone graft; bone surgery/joint surgery/arthroplasty; bursitis unspecified/infected bursa; cervical spondylosis/neck arthritis; charcot joint/neuropathic joint/neuropathic foot; connective tissue disease; disorder of the bone; elbow joint surgery; foot surgery; frozen shoulder; ganglion removal; giant cell arteritis/temporal arteritis; gout; haemarthrosis; hand surgery; hip joint surgery; hip replacement; jaw/temporomandibular surgery/cheek bone surgery; joint aspiration or injection; joint dislocation; joint disorder/joint pain; knee arthroplasty/ wash out; knee joint surgery; knee replacement; manipulation of joint; meniscectomy of the knee/removal or repair of semilunar cartilages; microscopic polyarteritis; muscle investigations/muscle biopsy; muscle problem; neck surgery; operation on bursa; orthopaedic or joint investigations; osteoarthritis; osteoporosis; other musculoskeletal problem/procedure; Paget's disease; polymyalgia rheumatica; prolapsed intervertebral disc/slipped disc; release of Dupuytren's contracture; rheumatoid arthritis; scleroderma; septic arthritis/infected joint; shoulder dislocation; shoulder joint surgery; Sjogren's syndrome/sicca syndrome; soft tissue problem; soft tissue surgery; spine arthritis/spondylosis; spine or back surgery; systemic lupus erythematosis/SLE; temporal artery biopsy; tendon operation; vasculitis; Wegener's granulomatosis; wrist surgery |
| Any haematological SAE | Anaemia; anaemia investigations/blood loss investigation; aplastic anaemia; blood transfusion; bone marrow biopsy; clotting disorder/excess bleeding; hereditary/genetic haematological disorder; INR problem/anti-coagulation problem or stabilisation; iron deficiency anaemia; monoclonal gammopathy of uncertain significance (MGUS); myelodysplasia or myelofibrosis; myeloproliferative disorder; neutropenia/lymphopenia; pancytopenia; pernicious anaemia; polycythaemia (primary or secondary); pure red cell aplasia; renal anaemia; thrombocytopenia/low platelets |
| Any ophthalmic SAE | Cataract extraction/lens implant; eye infection; eye investigations; eye surgery; eye trauma; eye/eyelid problem; eyelid and eyebrow operation; glaucoma (any type); glaucoma surgery/trabeculectomy; macular/posterior pole degeneration; nasolacrimal duct operation; operation on the vitreous body/vitrectomy; retinal artery occlusion/retinal thrombosis; retinal detachment; retinal or vitreous (or eye) haemorrhage; retinal problem; retinal vein occlusion; visual loss or deterioration/eyesight problem |
| Any ear, nose and throat SAE | Biopsy of the pharynx/nasopharynx/throat; ear disorder; ear drum repair/tympanic membrane surgery; ear infection/otitis media or externa; ear lesion biopsy/removal; ear surgery; laryngeal/vocal cord biopsy; laryngoscopy; larynx surgery; mastoidectomy/mastoid surgery; mouth problem/procedure; nasal cautery/nosebleed treatment; nasal polypectomy; nasal surgery; nose bleed/epistaxis; nose disorder; oral biopsy; parotid gland infection; parotid gland problem/surgery; perforated ear drum; salivary gland problem/surgery; sinus problem/surgery; throat surgery/pharynx surgery; tooth extraction/dental procedure; uvula surgery/snoring surgery/uvulopalatopharyngoplasty; vertigo or vestibular disorder |
| Any endocrinology SAE |  |
| Diabetes related complications | Diabetic coma unspecified; diabetic eye disease; diabetic foot, toe or leg ulcer; diabetic ketoacidosis/ ketoacidotic coma; diabetic non ketotic hyperosmolar state/coma; laser treatment for diabetic eye disease /photocoagulation; unstable diabetes/stabilisation of diabetes/hyperglycaemia; hypoglycaemia/low sugar/hypoglycaemic coma |
| Other endocrine disorder | Adrenal adenoma/tumour; adrenocortical insufficiency/Addison's disease; Cushing's syndrome; disorder of adrenal gland; endocrine investigations; hyperaldosteronism/Conn's syndrome; hyperthyroidism/thyrotoxicosis; hypothyroidism/myxoedema; other endocrine problem; thyroid problem; thyroid surgery |
| Any other medical | Abscess/drainage of abscess; adjustment of medication or treatment; admitted for investigation; admitted for surgery or procedure but not done; allergic or anaphylactic reaction to drug; allergic or anaphylactic reaction to food; allergy/hypersensitivity/anaphylaxis; amyloid (other/unknown); bacterial infection; block dissection of nodes/ node excision; chest pain/tightness; collapse/vasovagal attack/syncope; cytomegalovirus (CMV); death; death out-of-hospital; dehydration; dengue fever; faintness/lightheadedness/presyncope; fall/collapse; fever/pyrexia of unknown origin; flu like symptoms/influenza; fluid/electrolyte/acid-base imbalance; fungal infection; fungal skin or mouth infection; glandular fever/infectious mononucleosis; hospital admission reason unclear/insufficient details; hypernatraemia; hyponatraemia; infection; investigation of weight loss; legionnaires disease; lymph node biopsy; Methicillin resistant Staphylococcus aureus (MRSA); multi-organ failure; nephrogenic systemic sclerosis; oesophageal or intestinal candida; pain relief/pain management; peripheral oedema; post stroke complications; post-operative check/procedure; post-operative complication/post procedure complication; post-operative haemorrhage/haematoma; post-operative infection; post-operative pain; post-operative wound problem; preoperative assessment; primary (al) amyloid; protozoal infection; reduced mobility; rehabilitation/respite care/convalescence; removal of wire from chest wall; secondary amyloid; septicaemia; shingles/chicken pox/varicella-zoster infection; sudden death; systemic/invasive fungal infection; terminal care/terminally ill/palliative care; therapeutic epidural/spinal injection or nerve block; tuberculosis; typhoid fever/paratyphoid fever/enteric fever; unspecified biopsy; unspecified tumour removal/surgery; upper respiratory tract infection; viral infection |
|  |  |
| **Trauma/fracture** |  |
| Any trauma SAE | Accident; accidental poisoning; animal/insect/spider bite; ankle/foot fracture; assault; bone fracture; bone fracture procedure; chest injury; clavicle/collar bone/shoulder/scapular fracture; colles fracture/fracture wrist; external fixation of fracture/adjustment of external fixation device; fracture neck of femur/hip fracture; fracture rib or sternum; fracture tibia and/or fibula; fracture upper arm/humerus fracture/elbow fracture; hand/finger/thumb fracture; head injury; heat stroke/sun stroke; hypoxic brain damage; injury; internal fixation of fracture/adjustment of internal fixation device; internal injury; neurological injury/trauma; post-traumatic wound infection; reduction of fracture; road traffic accident; shaft femur fracture; skull/jaw/facial bone fracture; soft tissue injury to arm or hand injury; soft tissue injury to back, trunk or chest; soft tissue injury to hip or thigh or buttock; soft tissue injury to leg or foot; soft tissue/superficial injury; spinal cord injury; traumatic intracranial haemorrhage; vertebral fracture/crush fracture/vertebral collapse |
|  |  |
| ***Vascular SAEs*** |  |
| Any vascular SAE | Above knee amputation; acute coronary syndrome/ hospitalisation with angina; acute ischaemic limb; amaurosis fugax/transient visual loss; amputation of finger/thumb; amputation of foot; amputation of toe; angiogram of leg/femoral angiogram; Aortic aneurysm; Aortic aneurysm dissection; aortic aneurysm repair or stent; Aortic aneurysm rupture; aortic valve repair/replacement; arrhythmia; arterial embolism/thrombosis; arterial graft reconstruction/excision (not dialysis access); arterial surgery (not dialysis access); atrial fibrillation/flutter; below knee amputation; bradycardia; cardiac arrest; cardiac congestion of liver; cardiac death; cardiomyopathy; cardiovascular investigations; cardioversion; carotid angiogram or arch aortogram; carotid angioplasty +/- stent; carotid surgery; cerebral artery aneurysm surgery or clipping; CHD death (not MI); conduction disorder/heart block; conduction system ablation; cor pulmonale or right heart failure; coronary angiogram/cardiac catheterisation; coronary angioplasty (PTCA) +/- stent; coronary artery bypass graft (CABG); deep vein thrombosis (DVT); electrophysiological studies (EPS); embolectomy; embolism/thrombosis arm artery; embolism/thrombosis leg artery; fem-pop bypass/leg artery bypass; haemorrhagic stroke; heart failure - ischaemic; heart failure - not ischaemic; heart failure/pulmonary oedema/congestive cardiac failure; heart transplant; heart valve problem; heart valve surgery; hypotension; infected arterial graft (not dialysis access); infective endocarditis/subacute bacterial endocarditis; internal cardiac defibrillator insertion/problem/battery change or check; ischaemic/ gangrenous toe or finger; ischaemic/non-haemorrhagic stroke; ischaemic/non-haemorrhagic stroke - definite; Ischaemic/non-haemorrhagic stroke - presumed; leg artery angioplasty +/- stent; ligation/stripping/injection of varicose veins; limb ischaemia; mitral valve repair/replacement; myocardial infarction - definite; myocardial infarction - possible; myocardial infarction - probable; non coronary angiogram; non coronary angioplasty +/- stent; non coronary arterial surgery/intervention (not dialysis access); occluded arterial graft (not dialysis access); operation on/infection in/problem with amputation stump; other cardiac death (not CHD); other cardiovascular procedures; pacemaker insertion/change/battery change; palpitations/fluttering of heart; pericardial effusion; pericardial surgery/pericardial drainage; pericarditis including Dressler's; popliteal or femoral or iliac aneurysm repair; postural hypotension; pulmonary embolism (PE) +/- DVT; renal artery angiogram; renal artery angioplasty +/- stent; spontaneous subdural haematoma; stroke; subarachnoid haemorrhage; sudden cardiac death; supraventricular tachycardia (SVT); tachycardia; transient ischaemic attack (TIA) (neurological symptoms < 24 hours); uncontrolled hypertension/ stabilisation of blood pressure; venogram; venogram central; ventricular tachycardia (VT) |
