# Supplementary material for: Effect on non-vascular outcomes of lowering LDL cholesterol in patients with chronic kidney disease: results from the Study of Heart and Renal Protection
Source: BMC Nephrol. 2017 May 1;18:147. doi: 10.1186/s12882-017-0545-2 (PMC5412040; doi:10.1186/s12882-017-0545-2)
Supplement: Supplementary file 2 — All non-vascular SAEs, by particular baseline characteristics. Figure S2. All non-vascular SAEs, by system of disease and dialysis status. Figure S3. All non-vascular SAEs, by duration of follow-up. (PDF 21 kb) [file 12882_2017_545_MOESM2_ESM.pdf]

Webfigure 1: All non−vascular SAEs, by particular baseline characteristics

	Simvastatin plus ezetimibe (n=4650)	Placebo (n=4620)	Risk ratio (95% CI)	p value
**Age (χ^2_1=0.00, p=1.00)**				
40−49	744/968 (76.9%)	698/908 (76.9%)	1.02 (0.92−1.13)	
50−59	845/1161 (72.8%)	847/1149 (73.7%)	0.96 (0.87−1.06)	
60−69	938/1226 (76.5%)	957/1246 (76.8%)	1.01 (0.92−1.10)	
≥70	1024/1295 (79.1%)	1035/1317 (78.6%)	1.00 (0.91−1.09)	
**Sex (χ^2_1= 0.00, p=0.97)**				
Male	2196/2915 (75.3%)	2170/2885 (75.2%)	0.99 (0.94−1.06)	
Female	1355/1735 (78.1%)	1367/1735 (78.8%)	1.00 (0.92−1.07)	
**Diabetes (χ^2_1=0.01, p=0.94)**				
No diabetes	2699/3596 (75.1%)	2716/3580 (75.9%)	0.99 (0.94−1.05)	
Diabetes	852/1054 (80.8%)	821/1040 (78.9%)	1.00 (0.90−1.10)	
**Baseline LDL−cholesterol (χ^2_1=0.23, p=0.63)**				
<2.5	1389/1776 (78.2%)	1338/1707 (78.4%)	1.01 (0.94−1.09)	
≥2.5 <3.0	811/1059 (76.6%)	792/1037 (76.4%)	1.01 (0.92−1.12)	
≥3.0	1209/1627 (74.3%)	1259/1686 (74.7%)	0.98 (0.91−1.06)	
**Body mass index (χ^2_1=0.21, p=0.65)**				
<24	1052/1385 (76.0%)	1036/1338 (77.4%)	0.97 (0.89−1.05)	
≥24 <28	1151/1523 (75.6%)	1135/1533 (74.0%)	1.03 (0.95−1.12)	
≥28	1274/1646 (77.4%)	1281/1652 (77.5%)	1.00 (0.92−1.08)	
**Ethnicity (χ^2_3=6.45, p=0.09)#**				
White	2624/3332 (78.8%)	2656/3314 (80.1%)	0.98 (0.92−1.03)	
Chinese	397/557 (71.3%)	360/563 (63.9%)	1.18 (1.03−1.36)	
Other Asian	307/486 (63.2%)	310/480 (64.6%)	0.95 (0.81−1.12)	
Black/other/not specified	223/275 (81.1%)	211/263 (80.2%)	0.97 (0.81−1.18)	
**Renal status (χ^2_1=0.16, p=0.69)**				
eGFR ≥30	676/1144 (59.1%)	615/1099 (56.0%)	1.09 (0.98−1.21)	
eGFR ≥15 <30	914/1246 (73.4%)	1007/1319 (76.3%)	0.92 (0.84−1.01)	
eGFR <15	571/613 (93.1%)	560/606 (92.4%)	1.00 (0.89−1.12)	
On dialysis	1310/1534 (85.4%)	1280/1491 (85.8%)	1.00 (0.93−1.08)	
**All patients**	**3551/4650 (76.4%)**	**3537/4620 (76.6%)**	**0.99 (0.95−1.04)**	**0.82**

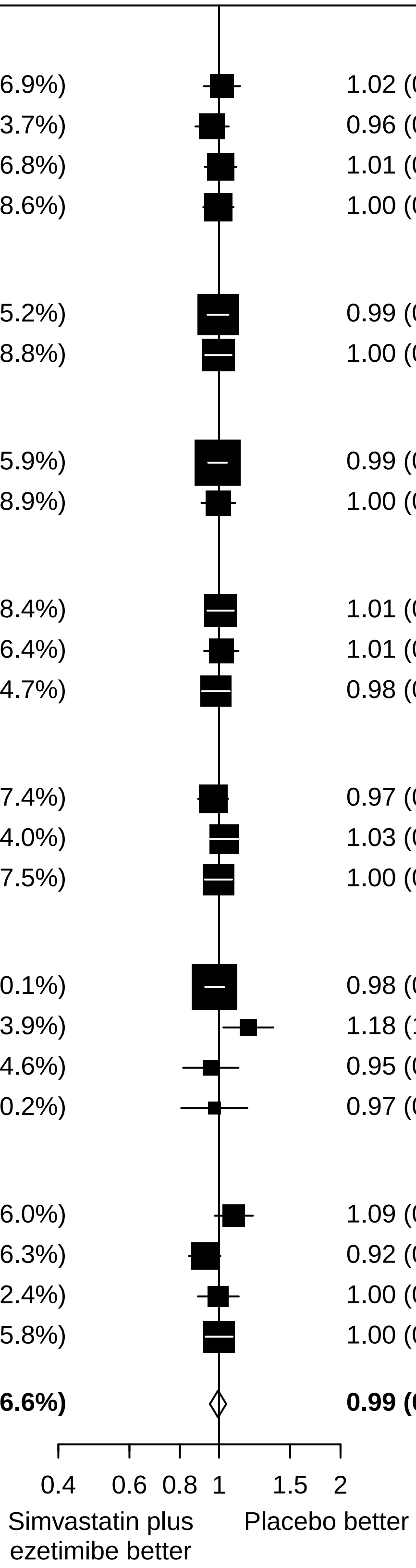

SAE=serious adverse event. eGFR = estimated glomerular filtration rate. #Subgroup analysis defined retrospectively.

Webfigure 2: All non−vascular SAEs, by system of disease and dialysis status

	Simvastatin plus ezetimibe (n=4650)	Placebo (n=4620)	Risk ratio (95% CI)	p value
**Cancer* (χ^2_1=0.11, p=0.74)**				
Not on dialysis	315/3116 (10.1%)	309/3129 (9.9%)	1.02 (0.87−1.20)	
On dialysis	151/1534 (9.8%)	152/1491 (10.2%)	0.98 (0.78−1.22)	
**All**	**466/4650 (10.0%)**	**461/4620 (10.0%)**	**1.01 (0.88−1.14)**	**0.94**
**Renal (χ^2_1=0.08, p=0.77)**				
Not on dialysis	1477/3116 (47.4%)	1497/3129 (47.8%)	0.98 (0.91−1.05)	
On dialysis	991/1534 (64.6%)	995/1491 (66.7%)	0.96 (0.88−1.05)	
**All**	**2468/4650 (53.1%)**	**2492/4620 (53.9%)**	**0.97 (0.92−1.03)**	**0.36**
**Respiratory (χ^2_1= 0.06, p=0.81)**				
Not on dialysis	399/3116 (12.8%)	405/3129 (12.9%)	0.99 (0.86−1.14)	
On dialysis	331/1534 (21.6%)	318/1491 (21.3%)	1.02 (0.87−1.19)	
**All**	**730/4650 (15.7%)**	**723/4620 (15.6%)**	**1.01 (0.91−1.12)**	**0.91**
**Hepatobiliary (χ^2_1=0.69, p=0.41)**				
Not on dialysis	95/3116 (3.0%)	102/3129 (3.3%)	0.93 (0.70−1.23)	
On dialysis	92/1534 (6.0%)	82/1491 (5.5%)	1.11 (0.82−1.49)	
**All**	**187/4650 (4.0%)**	**184/4620 (4.0%)**	**1.01 (0.83−1.24)**	**0.91**
**Other gastrointestinal (χ^2_1=0.49, p=0.49)**				
Not on dialysis	573/3116 (18.4%)	586/3129 (18.7%)	0.97 (0.87−1.09)	
On dialysis	415/1534 (27.1%)	435/1491 (29.2%)	0.92 (0.80−1.05)	
**All**	**988/4650 (21.2%)**	**1021/4620 (22.1%)**	**0.95 (0.87−1.04)**	**0.30**
**Other medical causes (χ^2_1=0.27, p=0.60)**				
Not on dialysis	1203/3116 (38.6%)	1196/3129 (38.2%)	1.01 (0.93−1.10)	
On dialysis	748/1534 (48.8%)	705/1491 (47.3%)	1.05 (0.94−1.16)	
**All**	**1951/4650 (42.0%)**	**1901/4620 (41.1%)**	**1.03 (0.96−1.09)**	**0.40**
**Trauma/fracture (χ^2_1= 0.18, p=0.67)**				
Not on dialysis	201/3116 (6.5%)	189/3129 (6.0%)	1.07 (0.88−1.31)	
On dialysis	164/1534 (10.7%)	160/1491 (10.7%)	1.01 (0.81−1.25)	
**All**	**365/4650 (7.8%)**	**349/4620 (7.6%)**	**1.05 (0.90−1.21)**	**0.54**
**Total: All non−vascular SAEs (χ^2_1= 0.07, p=0.79)**				
Not on dialysis	2241/3116 (71.9%)	2257/3129 (72.1%)	0.99 (0.93−1.05)	
On dialysis	1310/1534 (85.4%)	1280/1491 (85.8%)	1.00 (0.93−1.08)	
**All**	**3551/4650 (76.4%)**	**3537/4620 (76.6%)**	**0.99 (0.95−1.04)**	**0.82**

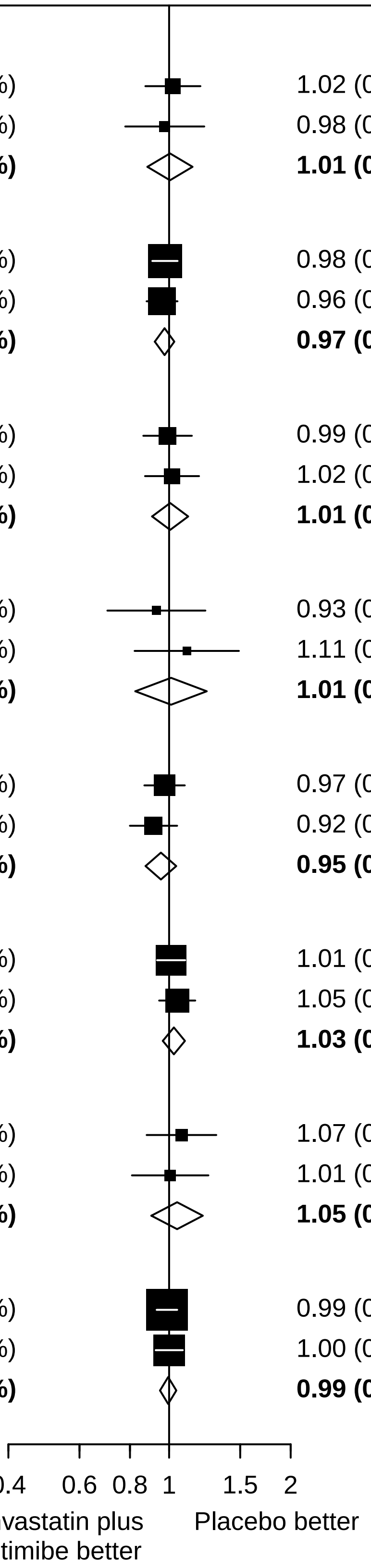

SAE=serious adverse event. *Includes cancer complications and deaths from pre−randomization cancers.

Webfigure 3: All non−vascular SAEs, by duration of follow−up

	Simvastatin plus ezetimibe (n=4650)	Placebo (n=4620)	Risk ratio (95% CI)	p value
**Non−vascular death (Trend: χ^2_1=1.69, p=0.19)**				
Year 1	111/4650 (2.4%)	109/4620 (2.4%)	1.01 (0.78−1.32)	
Year 2	134/4435 (3.0%)	137/4390 (3.1%)	0.97 (0.76−1.23)	
Year 3	153/4175 (3.7%)	136/4142 (3.3%)	1.12 (0.89−1.41)	
Year 4+	270/3825 (7.1%)	230/3829 (6.0%)	1.18 (0.99−1.41)	
**All follow−up**	**668/4650 (14.4%)**	**612/4620 (13.2%)**	**1.09 (0.98−1.21)**	**0.13**
**Non−fatal non−vascular SAEs (Trend: χ^2_1= 0.05, p=0.83)**				
Year 1	1706/4650 (36.7%)	1681/4620 (36.4%)	1.01 (0.94−1.08)	
Year 2	812/2794 (29.1%)	825/2786 (29.6%)	0.98 (0.89−1.08)	
Year 3	469/1907 (24.6%)	483/1898 (25.4%)	0.97 (0.85−1.10)	
Year 4+	493/1355 (36.4%)	489/1345 (36.4%)	1.01 (0.89−1.15)	
**All follow−up**	**3480/4650 (74.8%)**	**3478/4620 (75.3%)**	**1.00 (0.95−1.04)**	**0.87**
**Any non−vascular SAE**	**3551/4650 (76.4%)**	**3537/4620 (76.6%)**	**0.99 (0.95−1.04)**	**0.82**

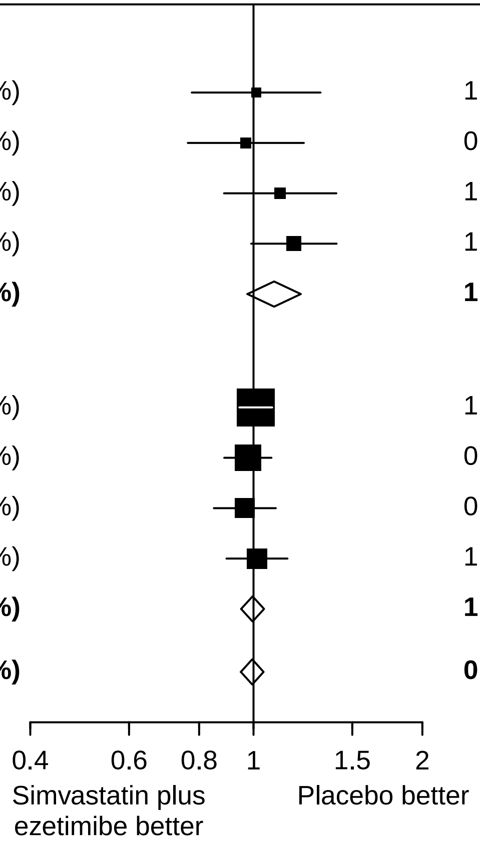

SAE=serious adverse event.
